# Early neonatal outcomes of very-low-birth-weight infants in Turkey: A prospective multicenter study of the Turkish Neonatal Society

**DOI:** 10.1371/journal.pone.0226679

**Published:** 2019-12-18

**Authors:** Esin Koc, Nihal Demirel, Ahmet Yagmur Bas, Dilek Ulubas Isik, Ibrahim Murat Hirfanoglu, Turan Tunc, Fatma Nur Sari, Guner Karatekin, Ramazan Ozdemir, Huseyin Altunhan, Merih Cetinkaya, Beyza Ozcan, Servet Ozkiraz, Sebnem Calkavur, Kadir Serafettin Tekgunduz, Ayhan Tastekin, Ferda Ozlu, Banu Mutlu Ozyurt, Ahmet Ozdemir, Bilin Cetinkaya, Yasar Demirelli, Esad Koklu, Ulker Celik, Nuriye Tarakci, Didem Armangil, Emel Okulu, Fatma Narter, Birgul Mutlu, Mustafa Kurthan Mert, Ali Bulbul, Huseyin Selim Asker, Ozgun Uygur, Ilker Sait Uslu, Sabahattin Ertugrul, Cumhur Aydemir, Hasan Tolga Celik, Kazim Kucuktasci, Selda Arslan, Hacer Ergin, Aysegul Zenciroglu, Sadik Yurttutan, Aysen Orman, Oguz Tuncer, Beril Yasa, Betul Acunas, Sahin Takci, Zeynel Gokmen, Hilal Ozkan, Serdar Comert, Nuran Ustun, Mehmet Mutlu, Bilge Tanyeri Bayraktar, Leyla Bilgin, Funda Tuzun, Ozge Aydemir, Tugba Gursoy, Arzu Akdag, Asli Memisoglu, Emrah Can, Demet Terek, Serdar Beken, Ozden Turan, Nilufer Guzoglu, Rahmi Ors, Yusuf Kale, Berna Hekimoglu, Hakan Aylanc, Funda Eroglu, Suzan Sahin, Murat Konak, Dilek Sarici, Ilknur Kilic, Nilay Hakan

**Affiliations:** 1 Department of Neonatology, Gazi University Faculty of Medicine, Ankara,Turkey; 2 Department of Neonatology, Yildirim Beyazit University Faculty of Medicine, Ankara, Turkey; 3 Department of Neonatology, Etlik Zubeyde Hanim Women’s Health Teaching and Research Hospital, University of Health Sciences, Ankara, Turkey; 4 Neonatology Division, Memorial Hospital, Istanbul, Turkey; 5 Department of Neonatology Dr Zekai Tahir Burak Women’s Health Education and Research Hospital, University of Health Sciences, Ankara, Turkey; 6 Department of Neonatology, Zeynep Kamil Maternity and Children’s Training and Research Hospital, University of Health Sciences, Istanbul, Turkey; 7 Department of Neonatology, Inonu University, Faculty of Medicine, Malatya, Turkey; 8 Department of Neonatology, Necmettin Erbakan University, Meram Faculty of Medicine, Konya, Turkey; 9 Department of Neonatology, Kanuni Sultan Suleyman Training and Research Hospital, University of Health Sciences, Istanbul, Turkey; 10 Department of Neonatology, Konya Education and Research Hospital, University of Health Sciences, Konya, Turkey; 11 Neonatology Division, Medicalpark Hospital, Gaziantep, Turkey; 12 Department of Neonatology, Dr Behcet Uz Children’s Hospital, University of Health Sciences, Izmir, Turkey; 13 Department of Neonatology, Ataturk University, Faculty of Medicine, Erzurum, Turkey; 14 Department of Neonatology, Medipol University, Faculty of Medicine, Istanbul, Turkey; 15 Department of Neonatology, Cukurova University, Faculty of Medicine, Adana, Turkey; 16 Neonatology Division, Mersin State Hospital, Mersin, Turkey; 17 Department of Neonatology, Erciyes University, Faculty of Medicine, Kayseri, Turkey; 18 Department of Neonatology, Baskent University, Faculty of Medicine, Adana, Turkey; 19 Department of Neonatology, Erzurum Nenehatun Maternity Hospital, Erzurum, Turkey; 20 Neonatology Division, Megapark Hospital, Kahramanmaras, Turkey; 21 Neonatology Division, Denizli State Hospital, Denizli, Turkey; 22 Department of Neonatology, Dr. Faruk Sukan Maternity and Children's Hospital, Konya, Turkey; 23 Department of Neonatology, Yuksek Ihtisas University, Faculty of Medicine, Ankara, Turkey; 24 Department of Neonatology, Ankara University Faculty of Medicine, Ankara, Turkey; 25 Department of Neonatology, Kartal Lutfi Kirdar Education and Research Hospital, University of Health Sciences, Istanbul, Turkey; 26 Neonatology Division, Doruk Private Hospital, Bursa, Turkey; 27 Department of Neonatology, Numune Training and Education Hospital, University of Health Sciences, Adana, Turkey; 28 Department of Neonatology, Sisli Hamidiye Etfal Education and Research Hospital, University of Health Sciences, Istanbul, Turkey; 29 Neonatology Division, NCR International Hospital, Gaziantep, Turkey; 30 Department of Neonatology, Tepecik Training and Research Hospital, University of Health Sciences, Izmir, Turkey; 31 Department of Neonatology, Ondokuz Mayıs University, Faculty of Medicine, Samsun, Turkey; 32 Department of Neonatology, Dicle University Faculty of Medicine, Diyarbakır, Turkey; 33 Department of Neonatology, Bulent Ecevit University Faculty of Medicine, Zonguldak, Turkey; 34 Department of Neonatology, Hacettepe University Faculty of Medicine, Ankara, Turkey; 35 Neonatology Division, Denizli Saglik Hospital, Denizli, Turkey; 36 Department of Neonatology, Mustafa Kemal University Faculty of Medicine, Hatay, Turkey; 37 Department of Neonatology, Pamukkale University Faculty of Medicine, Denizli, Turkey; 38 Department of Neonatology, Dr Sami Ulus Maternity and Children’s Hospital, University of Health Sciences, Ankara, Turkey; 39 Department of Neonatology, Kahramanmaras Sutcu Imam University, Faculty of Medicine, Kahramanmaras, Turkey; 40 Department of Neonatology, Fırat University, Faculty of Medicine, Elazig, Turkey; 41 Department of Neonatology, Yuzuncu Yil University, Faculty of Medicine, Van, Turkey; 42 Department of Neonatology, Istanbul University, Istanbul Faculty of Medicine, Istanbul, Turkey; 43 Department of Neonatology, Trakya University Faculty of Medicine, Edirne, Turkey; 44 Department of Neonatology, Gaziosmanpasa University, Faculty of Medicine, Tokat, Turkey; 45 Department of Neonatology, Baskent University, Faculty of Medicine, Konya, Turkey; 46 Department of Neonatology, Uludag University, Faculty of Medicine, Bursa, Turkey; 47 Department of Neonatology, Suleymaniye Maternity, Research & Training Hospital, University of Health Sciences, Istanbul, Turkey; 48 Department of Neonatology, Medeniyet University, Faculty of Medicine, Istanbul, Turkey; 49 Department of Neonatology, Karadeniz Technical University, Faculty of Medicine, Trabzon, Turkey; 50 Department of Neonatology, Bezmialem University, Faculty of Medicine, Istanbul, Turkey; 51 Department of Neonatology, Umraniye Education and Research Hospital, University of Health Sciences, Istanbul, Turkey; 52 Department of Neonatology, Dokuz Eylul University Faculty of Medicine, Izmir, Turkey; 53 Department of Neonatology, Osmangazi University Faculty of Medicine, Eskisehir, Turkey; 54 Department of Neonatology, Koc University, Faculty of Medicine, Istanbul, Turkey; 55 Department of Neonatology, Bursa Dortcelik Children's Hospital, Bursa, Turkey; 56 Department of Neonatology, Marmara University, Faculty of Medicine, Istanbul, Turkey; 57 Department of Neonatology, Bagcilar Education and Research Hospital, University of Health Sciences, Istanbul, Turkey; 58 Department of Neonatology, Ege University, Faculty of Medicine, Izmir, Turkey; 59 Department of Neonatology, Acıbadem University, Faculty of Medicine, Istanbul, Turkey; 60 Department of Neonatology, Baskent University, Faculty of Medicine, Ankara, Turkey; 61 Department of Neonatology, Kirikkale University, Faculty of Medicine, Kirikkale, Turkey; 62 Neonatology Division, Medova Hospital, Konya, Turkey; 63 Department of Neonatology, Cengiz Gokcek Maternity and Children's Hospital, Gaziantep, Turkey; 64 Department of Neonatology, Trabzon Kanuni Education and Research Hospital, University of Health Sciences, Trabzon, Turkey; 65 Department of Neonatology, Onsekizmart University, Faculty of Medicine, Canakkale, Turkey; 66 Neonatology Division, Ankara Guven Hospital, Ankara, Turkey; 67 Department of Neonatology, Adnan Menderes University, Faculty of Medicine, Aydin, Turkey; 68 Department of Neonatology, Konya Selcuk University, Faculty of Medicine, Konya, Turkey; 69 Department of Neonatology, Kecioren Education and Research Hospital, University of Health Sciences, Ankara, Turkey; 70 Neonatology Division, Atasehir Kadikoy Sifa Hospital, Istanbul, Turkey; 71 Department of Neonatology, Mugla Sıtkı Kocman University Faculty of Medicine, Mugla, Turkey; University of New South Wales, AUSTRALIA

## Abstract

**Objective:**

To investigate the early neonatal outcomes of very-low-birth-weight (VLBW) infants discharged home from neonatal intensive care units (NICUs) in Turkey.

**Material and methods:**

A prospective cohort study was performed between April 1, 2016 and April 30, 2017. The study included VLBW infants admitted to level III NICUs. Perinatal and neonatal data of all infants born with a birth weight of ≤1500 g were collected for infants who survived.

**Results:**

Data from 69 NICUs were obtained. The mean birth weight and gestational age were 1137±245 g and 29±2.4 weeks, respectively. During the study period, 78% of VLBW infants survived to discharge and 48% of survived infants had no major neonatal morbidity. VLBW infants who survived were evaluated in terms of major morbidities: bronchopulmonary dysplasia was detected in 23.7% of infants, necrotizing enterocolitis in 9.1%, blood culture proven late-onset sepsis (LOS) in 21.1%, blood culture negative LOS in 21.3%, severe intraventricular hemorrhage in 5.4% and severe retinopathy of prematurity in 11.1%. Hemodynamically significant patent ductus arteriosus was diagnosed in 24.8% of infants. Antenatal steroids were administered to 42.9% of mothers.

**Conclusion:**

The present investigation is the first multicenter study to include epidemiological information on VLBW infants in Turkey. Morbidity rate in VLBW infants is a serious concern and higher than those in developed countries. Implementation of oxygen therapy with appropriate monitoring, better antenatal and neonatal care and control of sepsis may reduce the prevalence of neonatal morbidities. Therefore, monitoring standards of neonatal care and implementing quality improvement projects across the country are essential for improving neonatal outcomes in Turkish NICUs.

## Introduction

The survival rate of very-low-birth-weight (VLBW) infants increased from about 50% to 80% after the establishment of neonatal intensive care units (NICUs) in the early 1970s [1]. The increase in the survival rate of VLBW infants has been particularly evident over the last three decades owing to improvements in perinatal and neonatal intensive care, including use of antenatal steroids, surfactant, and novel mechanical ventilation therapies [1, 2]. However, the morbidity rate remains high among VLBW infants. Morbidities such as sepsis, necrotizing enterocolitis (NEC), bronchopulmonary dysplasia (BPD), intraventricular hemorrhage (IVH), and retinopathy of prematurity (ROP) develop in many VLBW infants during hospitalization [3, 4]. These morbidities lead to prolonged hospital stays, a risk of rehospitalization, and poor long-term outcomes.

In recent years, Turkey has developed programs to improve neonatal health and NICU care [5]. The rate of morbidity of VLBW infants is an important indicator of intensive care standards and the quality of NICU care. The medical outcomes of VLBW infants have been reported by different NICUs in Turkey [6–8]; however, no multicenter data regarding the morbidity of VLBW infants are available. In this study, we aimed to investigate the early neonatal outcomes of VLBW infants discharged from NICUs.

## Material and methods

The study was approved by the ethical review committee of Gulhane Faculty of Medicine (Number: 02530/2016), written informed consent was obtained from the parents and the refusal rate was nearly 1.9% (65 patients). The data were analyzed anonymously.

**Fig 1:**
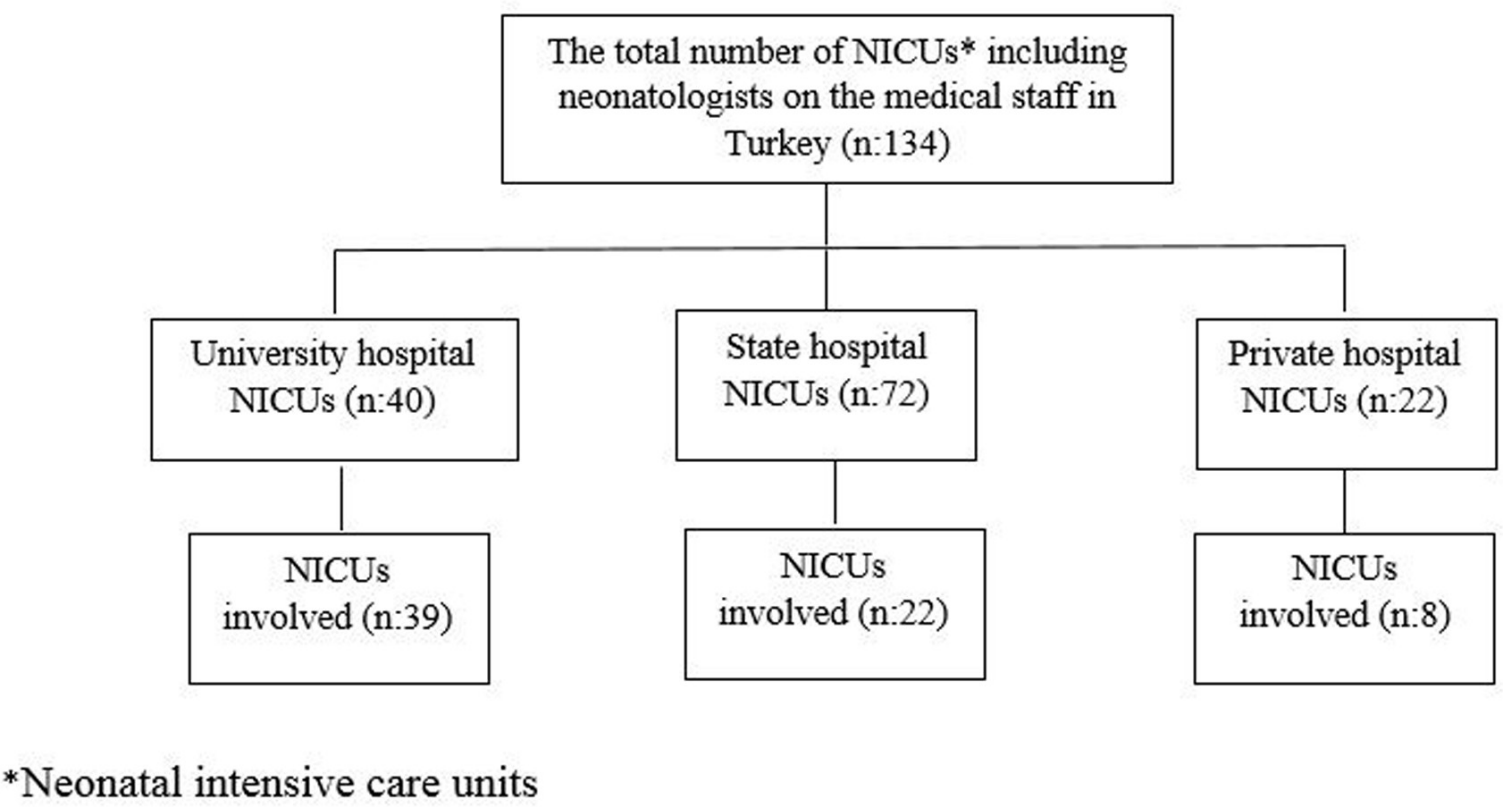
Types of NICUs involved in the study.

The present multicenter study included all VLBW infants admitted to level III NICUs comprising of neonatologists. The study was conducted between 1 April 2016 and 30 April 2017. In Turkey, the total number of NICUs including neonatologists on the medical staff is 134. In total, 69 NICUs agreed to take part in the study (51% of all) (Fig 1). Heads of the NICUs and directors of hospitals gave informed consent to participate in the research.

### Study population

Perinatal and neonatal data of all infants born with birth weight (BW) of ≤1500 g were collected until discharge home in survived infants. Delivery room deaths and infants who died during NICU care were not included because our study didn’t collect perinatal and neonatal data of these infants. All reported incidence proportions are on the basis of survivors only. This study also evaluated the prevalence of early neonatal outcomes of survived preterm infants born ≤32 weeks of gestation including infants > 1500 g birthweight, in a separate analysis.

The present study was promoted by the Turkish Neonatal Society. Data were collected through an online data entry system via a special network named the ‘Trials-Network’. A case report form (CRF) for each enrolled patient was completed by the participating neonatologist. All the questions in the CRF were required to be answered and “unknown” was a possible entry for some questions. The data entry system did not allow the collaborator to proceed and submit the data if no response was received for any question in the CRF. Anonymous data were entered into password protected database to maintain confidentiality. The records of infants from 69 NICUs were pooled together and analyzed at the end of the study.

The infants were excluded if they had congenital anomalies and malformations (e.g., diaphragmatic hernia, gastroschisis, atresia of the gastrointestinal tract, meningomyelocele, hydrocephalus, chromosomal anomalies, and complex congenital heart disease) from the study. The infants who died before the hospital discharge were also excluded.

### Clinical characteristics

Antenatal and natal clinical data including maternal age, administration of antenatal corticosteroids, preeclampsia/eclampsia, infants of diabetic mothers, chorioamnionitis (clinical or histopathological), in vitro fertilization, multiple births and mode of delivery were recorded. Antenatal steroid therapy was considered to be given if mother received two doses of 12 mg of betamethasone intramuscularly 24 hours apart at any time prior to delivery.

Gestational age was determined as the best obstetric estimate using ultrasonography first trimester and/or date of last menstrual period. The clinical characteristics of infants including gender, gestational age (GA), BW, small for gestational age (SGA; BW< 10th percentile for gender), resuscitation in the delivery room, respiratory distress syndrome (RDS), surfactant treatment, duration of invasive/noninvasive mechanical ventilation, oxygen therapy, hemodynamically significant patent ductus arteriosus (PDA), and major morbidities were also recorded on the CRF for each patient.

The major morbidities were defined as severe IVH (> Grade II according to Papile staging) [9], NEC (≥Stage II in accordance with the modified Bell criteria) [10], BPD (supplemental oxygen requirement at 36 weeks’ postmenstrual age) and severe ROP (requiring treatment). Late onset sepsis (LOS) was defined as the onset of symptoms at >72 hours of age. Patients with systemic signs of infection as well as positive blood cultures were diagnosed as culture proven LOS and those with negative cultures were considered as culture negative LOS [11]. Survival to discharge home and survival without major morbidity were determined.

### Statistical analysis

The data were collected and analyzed using the SPSS version 17.0 (SPSS Inc., Chicago, IL). The data were presented as n/N (%) for categorical variables, and as mean±standard deviation, for numeric variables. The nonparametric Kruskal–Wallis analysis was used to compare differences between the hospital groups for birth weight. Pair-wise comparisons were performed using Bonferroni adjusted Mann–Whitney U tests. The Chi-square test were performed to determine the statistical significance for administration of antenatal steroids and early neonatal outcomes among NICUs in university, state and private hospitals. A p-value of less than 0.05 was considered significant for the statistical tests.

## Results

The number of live born VLBW infants admitted to neonatal care was 4335 in participating 69 NICUs during the study period, excluding delivery room deaths. The mortality was 22% during NICU care. The study included 3381 VLBW infants at discharge home.

Perinatal and neonatal data from NICUs of 39 university hospitals (n = 1617), 22 state hospitals (n = 1433), and eight private hospitals (n = 331), were obtained. The mean BW and GA were 1137±245 g and 29±2.4 weeks, respectively. The BW of 1037 (30.6%) infants was less than 1000 g and 1430 (42.3%) infants were less than 28 weeks of gestation. There were 1791 (53%) females and 1590 (47%) males in the study. During the study period, 78% of VLBW infants survived to discharge and 48% of survived infants had no major neonatal morbidity.

The perinatal baseline characteristics and outcomes of discharged VLBW infants are presented in Table 1. Antenatal steroids were administered to 42.9% of mothers. Twenty-six percent of mothers had preeclampsia, 5.6% had gestational diabetes, and 9.8% had chorioamnionitis. The rate of cesarean delivery was 86.8% and the prevalence of multiple births was 24.7%. The infants were evaluated in terms of early neonatal outcomes: hemodynamically significant PDA was detected in 24.8% of infants, BPD in 23.7%, NEC in 9.1%, blood culture proven LOS in 21.1%, blood culture negative LOS in 21.3% and severe IVH in 5.4%. The incidence of severe ROP was 11.1%. The median GA of infants with severe ROP was 27 weeks (IQR 25–28) and median BW was 860 g (IQR 720–1040).

**Table 1:** Perinatal baseline characteristics and outcomes of discharged infants with BW ≤ 1500 g.

Characteristics	BW ≤500 g (n = 6)	BW 501–750 g (n = 270)	BW 751–1000 g (n = 761)	Subtotal BW≤1000g (n = 1037)	BW 1001–1250 g (n = 1060)	BW 1251–1500 g (n = 1284)	BW Total ≤ 1500 g (n = 3381)
Antenatal steroid, two doses, n (%)	1 (16.7%)	117 (43.3%)	351 (46.1%)	469 (45.2%)	467 (44%)	514 (40%)	1450 (42.9%)
Preeclampsia, n (%)	1 (16.7%)	100 (37%)	217 (28.5%)	318 (30.6%)	288 (27.2%)	283 (22%)	889 (26.3%)
Gestational diabetes, n (%)	1 (16.7%)	14 (5.2%)	45 (5.9%)	60 (5.8%)	60 (5.7%)	71 (5.5%)	191 (5.6%)
Chorioamnionitis, n (%)	-	18 (6.7%)	107 (14%)	125 (12%)	95 (9%)	113 (8.8%)	333 (9.8%)
IVF pregnancy, n (%)	1 (16.7%)	32 (11.8%)	81 (10.6%)	114 (11%)	115 (10.8%)	151 (11.8%)	380 (11.2%)
Multiple births							
Twin, n (%)	1 (16.7%)	41 (15.2%)	124 (16.3%)	166 (16%)	201 (19%)	322 (25.1%)	689 (20.4%)
Triplet, n	-	5 (1.8%)	35 (4.6%)	40 (3.9%)	47 (4.4%)	59 (4.6%)	146 (4.3%)
Vaginal delivery, n (%)	-	38 (14%)	109 (14.3%)	147 (14.2%)	120 (11.3%)	181 (14.1%)	448 (13.2%)
Gestational age (weeks)*	25.3±2.2	26.1±2	27.5 ± 1.9	27±2	29.1±1.9	30.5±1.8	29±2.4
Birth weight, (grams)*	444±41	665±65	898 ± 74	835±128	1132 ± 71	1386 ± 71	1137 ± 245
Male gender, n (%)	3 (50%)	113 (41.8%)	343 (45.1%)	459 (44.3%)	513 (48.4%)	630 (49.1%)	1602 (47.4%)
SGA, n (%)	6 (100%)	108 (40%)	184 (24.2%)	298 (28.8%)	232 (21.9%)	222 (17.3%)	752 (22.2%)
Resuscitation at birth, n (%)	5 (83.3%)	223 (82.6%)	513 (67.4%)	741 (71.4%)	536 (50.6%)	494 (38.5%)	1771 (52.4%)
RDS, n (%)	6 (100%)	255 (94.4%)	627 (82.4%)	888 (85.7%)	763 (71.2%)	712 (55.4%)	2363 (69.9%)
Surfactant treatment, n (%)	6 (100%)	241 (89.2%)	565 (74.2%)	812 (78.3%)	622 (58.7%)	534 (41.6%)	1968 (58.2%)
Duration of invasive mechanical ventilation (days)**	3 (0–48)	14 (2–42)	3 (0–15)	5 (0–21)	1 (0–4)	0 (0–2)	1 (0–6)
Duration of noninvasive ventilation (days)**	26 (7–89)	19 (10–30)	12 (4–24)	14 (5–26)	5 (2–12)	3 (1–6)	5 (2–14)
Total days on oxygen (days)**	54 (26–135)	68 (40–95)	39 (15–64)	47 (20–75)	18 (7–41)	8 (4–20)	19 (6–46)
PDA requiring treatment, n (%)	4 (66.7%)	139 (51.5%)	276 (36.3%)	419 (40.4%)	238 (22.4%)	180 (14%)	837 (24.8%)
Intracranial hemorrhage (> Grade II), n (%)	-	39 (14.4%)	50 (6.6%)	89 (8.6%)	49 (4.7%)	46 (3.6%)	184 (5.4%)
Late-onset sepsis, n (%)	5 (83.3%)	220 (81.5%)	451 (59.3%)	676 (65.2%)	421 (39.8%)	338 (26.3%)	1435 (42.4%)
Culture proven, n (%)	2 (33,3%)	119 (44.1%)	235 (30.9%)	356 (34.3%)	202 (19%)	157 (12.2%)	715 (21.1%)
NEC (≥ Stage II), n (%)	1 (16.7%)	45 (16.7%)	96 (12.6%)	142 (13.7%)	94 (8.9%)	70 (5.4%)	306 (9.1%)
BPD, n (%)	4 (66.7%)	187 (69.3%)	285 (37.4%)	476 (45.9%)	205 (19.3%)	119 (9.3%)	800 (23.7%)
Any degree of ROP, n (%)	3 (50%)	224 (82.9%)	507 (66.6%)	734 (70.8%)	428 (40.4%)	267 (20.8%)	1429 (42.3%)
Severe ROP, n (%)	1 (16.7%)	120 (44.4%)	151 (19.8%)	272 (26.2%)	73 (6.9%)	32 (2.5%)	377 (11.1%)

*Values are presented as mean±SD

** Values are presented as median, and IQR (interquartile range) are given in parenthesis

BW: Birth weight, IVF: Invitro fertilization, SGA:Small for gestational age, RDS:Respiratosy distress syndrome, PDA: Patent ductus arteriosus, NEC:Necrotizing enterocolitis, BPD:Bronchopulmonary dysplasia, ROP: Retinopathy of prematurity

Table 2[]shows the perinatal baseline characteristics and outcomes of discharged infants with a GA ≤32 weeks; hemodynamically significant PDA was detected in 20.2% of infants, BPD in 19.1%, NEC in 8.4%, blood culture proven LOS in 18.2%, severe IVH in 4.7% and severe ROP in 8.2%.

**Table 2:** Perinatal baseline characteristics and outcomes of discharged infants with GA ≤ 32 weeks.

Characteristics	GA 22–24 weeks (n = 87)	GA 25–26 weeks (n = 434)	GA 27–28 weeks (n = 968)	Subtotal GA≤28 weeks (n = 1489)	GA 29–30 weeks (n = 1454)	GA 31–32 weeks (n = 1906)	GA Total ≤ 32 weeks (n = 4849)
Antenatal steroid, two doses, n (%)	34 (39.1%)	182 (41.9%)	398 (41.1%)	614 (41.2%)	580 (39.9%)	738 (38.7%)	1932 (39.8%)
Preeclampsia, n (%)	9 (10.3%)	76 (17.5%)	220 (22.7%)	305 (20.5%)	332 (22.8%)	399 (20.9%)	1036 (21.4%)
Gestational diabetes, n (%)	2 (2.3%)	22 (5.1%)	63 (6.5%)	87 (5.8%)	88 (6%)	137 (7.2%)	312 (6.4%)
Chorioamnionitis, n (%)	15 (17.2%)	65 (15%)	128 (13.2%)	208 (14%)	133 (9.1%)	140 (7.3%)	481 (10%)
IVF pregnancy, n (%)	17 (19.5%)	36 (8.3%)	106 (11%)	159 (10.7%)	154 (10.6%)	215 (11.3%)	528 (10.9%)
Multiple births							
Twin, n (%)	20 (23%)	60 (13.8%)	202 (20.9%)	282 (18.9%)	316 (21.7%)	550 (28.8%)	1148 (23.7%)
Triplet, n (%)	1(1.1%)	15 (3.4%)	34 (3.5%)	50 (3.3%)	64 (4.4%)	94 (4.9%)	208 (4.3%)
Vaginal delivery, n (%)	23 (26.4%)	96 (22.1%)	157 (16.2%)	276 (18.5%)	226 (15.5%)	271 (14.2%)	773 (15.9%)
Gestational age (weeks)*	23.5±0.6	25.6±0.4	27.6±0.5	26.8±1.3	29.6±0.5	31.5±0.5	29.5±2.1
Birth weight, (grams)*	683±123	853±163	1078 ± 223	987±241	1345 ± 281	1626 ± 329	1345 ± 392
Male gender, n (%)	43 (49.4%)	213 (49.1%)	497 (51.3%)	753 (50.6%)	787 (54.1%)	971 (50.9%)	2511 (51.8%)
SGA, n (%)	6 (6.9%)	108 (24.9%)	184 (19%)	298 (20%)	232 (15.9%)	222 (11.6%)	752 (15.5%)
Resuscitation at birth, n (%)	84 (96.5%)	329 (75.8%)	606 (62.6%)	1019 (68.4%)	651 (44.8%)	645 (33.8%)	2315 (47.7%)
RDS, n (%)	87 (100%)	397 (91.5%)	811 (83.8%)	1295 (87%)	969 (66.6%)	935 (49%)	3199 (66%)
Surfactant treatment, n (%)	85 (97.7%)	373 (85.9%)	715 (73.9%)	1173 (78.8%)	745 (51.2%)	651 (34.1%)	2569 (53%)
Duration of invasive mechanical ventilation (days)**	40 (13–56)	10 (1–30)	2 (0–9)	4 (0–18)	0 (0–3)	0 (0–1)	0(0–4)
Duration of noninvasive ventilation (days)**	22(10–31)	17(7–30)	8(4–18)	11 (4–35)	4 (2–8)	2 (1–5)	4 (2–10)
Total days on oxygen (days)**	80 (57–114)	60(38–84)	33 (12–52)	42(19–66)	11 (5–27)	6 (2–12)	12 (5–35)
PDA requiring treatment, n (%)	60 (69%)	216 (49.8%)	320 (33%)	596 (40%)	233 (16%)	152 (8%)	981 (20.2%)
Intracranial hemorrhage (> Grade II), n (%)	15 (17.2%)	56 (12.9%)	66 (6.8%)	137 (9.2%)	49 (3.4%)	41 (2.1%)	227 (4.7%)
Late-onset sepsis, n (%)	75 (86.2%)	304 (70%)	474 (49%)	853 (57.3%)	508 (34.9%)	433 (22.7%)	1794 (37%)
Culture proven, n (%)	37 (42.5%)	154 (35.5%)	245 (25.3%)	436 (29.3%)	237 (16.3%)	209 (11%)	882 (18.2%)
NEC (≥ Stage II), n (%)	22 (25.3%)	68 (15.7%)	117 (12.1%)	207 (13.9%)	109 (7.5%)	92 (4.8%)	408 (8.4%)
BPD, n (%)	65 (74.7%)	239 (55.1%)	295 (30.5%)	599 (40.2%)	208 (14.3%)	121 (6.3%)	928 (19.1%)
Any degree of ROP, n (%)	68 (78.2%)	350 (80.6%)	526 (54.3%)	944 (63.4%)	422 (29%)	230 (12.1%)	1596 (32.9%)
Severe ROP, n (%)	49 (56.3%)	151 (34.8%)	120 (12.4%)	320 (21.5%)	58 (4%)	18 (0.9%)	396 (8.2%)

GA: Gestational age, IVF: Invitro fertilization, SGA:Small for gestational age, RDS:Respiratory distress syndrome, PDA: Patent ductus arteriosus, NEC:Necrotizing enterocolitis, BPD:Bronchopulmonary dysplasia, ROP: Retinopathy of prematurity

*Values are presented as mean±standart deviation

** Values are presented as median, and IQR (interquartile range) are given in parenthesis

The incidence of early neonatal outcomes of VLBW infants among NICUs in private hospitals, state hospitals, and university hospitals is shown in Table 3. The rate of administration of antenatal steroids was 26% in private hospitals which is lower than that in state hospitals (39.8%) and in university hospitals (49%). The incidence of LOS (42% *vs* 32% and 38%), NEC (24.5% *vs* 6.4% and 8.2%), bronchopulmonary dysplasia (29.3% *vs* 19.5% and 25.9%) and severe ROP (13.9% *vs* 10.9% and 10.7%) were significantly higher in private hospitals as compared to state hospitals and university hospitals, respectively.

**Table 3:** The incidence of early neonatal outcomes of VLBW infants among NICUs in university hospitals, in state hospitals and in private hospitals.

	University Hospitals (n = 1617)	State hospitals (n = 1433)	Private hospitals (n = 331)	p-value
Birth weight, g (mean± SD)	1119±248^**A**^	1145±245 ^**A**^	1212±215 ^**B**^	<0.001
Antenatal steroid, two doses, %	49.0 ^**A**^	39.8 ^**B**^	26.0 ^**C**^	<0.001
PDA requiring treatment, %	24.9	24.8	24.6	0.756
Intracranial hemorrhage (>Grade II), %	5.7	5.4	4.2	0.566
Late onset sepsis, %	38 ^**A**^	32.0 ^**B**^	42.0 ^**C**^	<0.001
NEC (≥Stage II), %	8.2 ^**A**^	6.4 ^**A**^	24.5 ^**B**^	<0.001
Bronchopulmonary dysplasia, %	25.9 ^**A**^	19.5 ^**B**^	29.3 ^**A**^	<0.001
Severe ROP, %	10.7 ^**A**^	10.9 ^**A**^	13.9 ^**B**^	<0.001

PDA: Patent ductus arteriosus, NEC: Necrotizing enterocolitis, ROP: Retinopathy of prematurity

The different capital letters in each row indicate significant differences between the hospital groups.

## Discussion

This is the first multicenter study to evaluate the early neonatal outcomes of VLBW infants in Level III NICUs in Turkey. The preterm birth rate is estimated to be about 11% worldwide [12], compared to 15% in Turkey. Around 23000 infants are born at less than 32 gestational weeks each year according to the Turkish Ministry of Health and Public Health Institute of Turkey (unpublished data, 2015). Our study included outcomes of 4849 discharged infants with a GA ≤32 weeks. The rest of the babies born at less than 32 gestational weeks were cared in NICUs with no neonatologists or in NICUs including neonatologists but who didn’t agree to participate in the study.

Improvements in newborn intensive care have resulted in increased survival rates in preterm infants. The survival rate of VLBW infants is reported to be between 85–90% in developed countries [13,14]. However, studies from developing countries put the survival rate at between 66–74% [15–17]. During the current study period, 78% of VLBW infants survived to discharge in Turkish Level III NICUs.

Antenatal steroid administration reduces the risk of RDS, IVH, NEC, sepsis, and mortality in preterm infants [18]. In the present study, the rate of antenatal steroid administration was nearly 43%, where this ratio is dramatically lower than that in developed countries. According to reports from developed countries, the rate of antenatal steroid administration increased from 16% in the 1980s to 90% in the 2010s among mothers of VLBW infants [14,19]. The lower rate of antenatal steroid administration in Turkey might contribute to the low survival rate compared to developed countries.

The data in the present study showed that nearly 70% of VLBW infants developed RDS, and 83.3% of those infants were treated with surfactant. The rate of surfactant use ranges from 58–62% in developed countries [13,14]. It is reported that when early continuous positive airway pressure is administered, babies of 26–29 weeks’ gestation can be managed without intubation or surfactant about 50% of the time [20]. Appropriate antenatal care, good obstetric practice, safe transportation of the newborn, optimal delivery room care, and the early application of non-invasive ventilation support strategies may decrease surfactant administration in RDS management in Turkey.

The incidence of BPD varies among institutions depending on a number of factors including intensive care practices and differences in the clinical definitions of BPD [21,22]. The BPD frequency is reported to be between 22–26% of VLBW infants in developed countries [13, 14]. In the present study, the incidence of BPD was 23.7% and 19.1% in infants with a BW ≤ 1500 g and GA ≤ 32 weeks, respectively. The rate of BPD in the present study was similar to the reported figures; however, the survival rate in VLBW infants was lower than in developed countries. Selective surfactant administration using less invasive procedures, prevention of infection by the use of control measures, and monitoring of targeted oxygen saturation are recommended to reduce the incidence of BPD [23, 24].

Late-onset sepsis is a common morbidity among preterm infants and is associated with poor neurodevelopmental outcomes and growth impairment [25]. The incidence of LOS is reported to be 15–25% of VLBW infants in developed countries [26, 27]. The frequency of culture-proven LOS in a Turkish study was 22% in VLBW infants[28]. In the present study, the incidence of LOS was 42.4%, of which culture-proven sepsis comprised half of the cases.

The reported incidence of NEC in preterm infants with a GA < 32 weeks varied from 2–7% among different centers in developed countries [29]. The incidence of advanced NEC in the present study was found to be 8.4% among infants with a GA ≤ 32 weeks. The incidence of sepsis and NEC could be reduced by infection control measures, including judicious use of antibiotic therapy, proper handwashing technique, increased awareness among healthcare staff, and avoidance of overcrowding. In addition, breastfeeding is one of the most effective practices for reducing NEC and sepsis in preterm infants[30].

Patent ductus arteriosus was reported in 39% of VLBW infants and 12% of VLBW infants required treatment [20]. In the present study, the rate of PDA requiring treatment was 24.8% in VLBW infants. Symptomatic PDA is common in preterm neonates, occurring in approximately 30% of VLBW infants [14]; however, there is currently no consensus among neonatologists on the management of PDA.

The incidence of severe IVH is approximately 7–15% in VLBW infants [14]. In the present study, the incidence of severe IVH was 5.4% and 4.7% in infants with a BW ≤1500 g and GA ≤ 32 weeks, respectively. IVH usually occurs within the first 72 hours of life and is an important cause of mortality in preterm infants. The characteristics of the population in the present study, which included surviving infants, might be the reason for the lower incidence of IVH.

Retinopathy of prematurity is a serious morbidity. In developed countries, the majority of infants born at > 28 weeks who develop ROP have mild disease that does not require treatment [31]. A nationwide population-based study from the UK revealed that the incidence of ROP requiring treatment was 4% in VLBW infants; the median GA at birth was 25 weeks and the median BW was 706 g[32]. In the present study, the rate of severe ROP was 11.1% in VLBW infants and the median BW and median GA in those infants were 860 g and 27 weeks, respectively. The findings of this study show that, in Turkey, more mature and heavier babies are at risk of severe ROP.

Although survival rates have improved, the incidence of major morbidities remains a serious concern. Major morbidity for VLBW survivors was reported to decrease from 46% in 2000 to 41% in 2009 in developed countries [27]. In another study, the survival rate of VLBW infants without major neonatal morbidity was found to be 70% [14]. In this study, 48% of survived infants had no major neonatal morbidity; this rate was lower than those in developed countries.

Turkey has universal health insurance system and the families do not have to pay for their babies’ NICU stay. All residents registered with the Social Security Institution can receive medical treatment free of charge in university, state and private hospitals. In the present study, the incidence of neonatal morbidities regarding LOS, BPD, severe ROP and especially NEC were significantly higher in private hospitals as compared to other facilities. This obvious variation between types of units are associated with less administration of antenatal steroids, prolonged antibiotic use, inappropriate monitoring of oxygen, lack of breast milk, and an assumed shortage of hospital expertise.

The strength of the present study was its large, multicenter design that allowed for prospective data collection via a specialized network. However, the neonatologists did not undertake any training to standardize the definitions of potential risk factors before the study began. In this article, we reported early neonatal outcomes in survived infants while the published data usually included results in all live births, this was another limitation of the study.

In Turkey, insufficient data on the prevalence of preterm morbidities hinders the establishment of strategies to minimize adverse outcomes. The present investigation is the first multicenter population-based study to include epidemiological information on VLBW infants. The results of this study will help predict, prevent, and improve adverse outcomes in VLBW infants. It also allows defining where we should allocate future efforts and resources for the benefit of most vulnerable newborns.

The current study provides an important overview for the Turkish population and for the entire world community as a comparator. Morbidity rate in VLBW infants is a serious concern in Turkey and higher than those in developed countries. It is of paramount importance to act by extending the use antenatal corticosteroids from an obstetric perspective. From the neonatal point of view, implementation of oxygen therapy with appropriate monitoring, better neonatal care, meticulous attention to hygiene procedures, control of sepsis and training NICU health care professionals may reduce the prevalence of neonatal morbidities.

In conclusion, monitoring standards of antenatal and neonatal care and implementing quality improvement projects across the country are essential for improving neonatal outcomes in Turkish NICUs.
